# Peripheral nervous system insulin resistance in *ob/ob* mice

**DOI:** 10.1186/2051-5960-1-15

**Published:** 2013-05-10

**Authors:** Caleb W Grote, Anna L Groover, Janelle M Ryals, Paige C Geiger, Eva L Feldman, Douglas E Wright

**Affiliations:** 1 Department of Anatomy and Cell Biology, the University of Kansas Medical Center, Kansas City, KS 66160, USA; 2 Department of Molecular and Integrative Physiology, the University of Kansas Medical Center, Kansas City, KS 66160, USA; 3 Department of Neurology, University of Michigan, Ann Arbor, MI 48109, USA

**Keywords:** Diabetic neuropathy, Neuronal insulin resistance, Neurotrophic support

## Abstract

**Background:**

A reduction in peripheral nervous system (PNS) insulin signaling is a proposed mechanism that may contribute to sensory neuron dysfunction and diabetic neuropathy. Neuronal insulin resistance is associated with several neurological disorders and recent evidence has indicated that dorsal root ganglion (DRG) neurons in primary culture display altered insulin signaling, yet *in vivo* results are lacking. Here, experiments were performed to test the hypothesis that the PNS of insulin-resistant mice displays altered insulin signal transduction *in vivo*. For these studies, nondiabetic control and type 2 diabetic *ob/ob* mice were challenged with an intrathecal injection of insulin or insulin-like growth factor 1 (IGF-1) and downstream signaling was evaluated in the DRG and sciatic nerve using Western blot analysis.

**Results:**

The results indicate that insulin signaling abnormalities documented in other “insulin sensitive” tissues (i.e. muscle, fat, liver) of *ob/ob* mice are also present in the PNS. A robust increase in Akt activation was observed with insulin and IGF-1 stimulation in nondiabetic mice in both the sciatic nerve and DRG; however this response was blunted in both tissues from *ob/ob* mice. The results also suggest that upregulated JNK activation and reduced insulin receptor expression could be contributory mechanisms of PNS insulin resistance within sensory neurons.

**Conclusions:**

These findings contribute to the growing body of evidence that alterations in insulin signaling occur in the PNS and may be a key factor in the pathogenesis of diabetic neuropathy.

## Background

Diabetes and metabolic syndrome are risk factors for several neurological diseases, and emerging evidence has indicated that neuronal insulin resistance may be involved in disease pathogenesis [1]. While altered insulin signaling is known to be the key factor in the development of diabetes, the role that it plays in diabetic neuropathy (DN) is not well understood. However, it has been demonstrated that neuronally-targeted insulin treatment can improve signs of neuropathy without altering blood glucose levels [2-4]. Recent evidence suggests that cultured sensory neurons from insulin-resistant mice display classic signs of insulin resistance and that insulin resistance may be contributing to mitochondrial dysfunction and increased ROS in DN [5-7]. Furthermore, clinical evidence has reported that insulin resistance appears to be an independent risk factor for both autonomic and peripheral neuropathy [8].

Although neurons do not appear to rely on insulin for glucose uptake [9], insulin does have an important role in both the CNS and PNS. Insulin promotes *in vivo* nerve regeneration [4,10,11], induces neurite outgrowth [12,13], maintains neuronal mitochondrial function [14,15], supports memory formation [16,17], and regulates hypothalamic metabolic control [18,19]. While the exact mechanisms through which insulin promotes these functions remain unclear, insulin is considered a potent neurotrophic factor key to maintaining proper neuronal function.

Insulin and insulin-like growth factor 1 (IGF-1) signaling is propagated by phosphorylation events that begin with the intrinsic tyrosine kinase activity of the insulin or IGF receptor (reviewed in [20,21]) and continue with subsequent activation of both the PI3K-Akt and MAPK cascades. While these pathways are well defined in muscle, adipose, and liver, insulin signaling and its actions in the PNS are poorly understood.

In an insulin-resistant state, the cellular effects of insulin are blunted due to improper signal propagation resulting from several different mechanisms, including 1) degradation of the insulin receptor [22-25], 2) removal of key tyrosine phosphorylation sites by overactivation of protein tyrosine phosphatases [26-29], and 3) increased phosphorylation at inhibitory IRS serine residues due to elevated stress kinases, such as JNK [30-35]. However, the extent to which these mechanisms affect insulin signal transduction in the PNS is not clear.

Growing evidence suggests that neurons may become insulin resistant similar to other tissues. However, no *in vivo* evidence of PNS insulin resistance has been presented, and the cellular mechanisms associated with PNS insulin resistance have not been thoroughly investigated. Here, we demonstrate that the DRG and sciatic nerve of *ob/ob* mice display reduced insulin signaling in response to an intrathecal injection of insulin. Furthermore, the PNS of *ob/ob* mice has alterations in cellular mechanisms of insulin resistance, including decreased DRG insulin receptor expression and upregulation of JNK activity in the sciatic nerve.

## Results

### Insulin resistance in *ob/ob* mice

**Figure 1:**
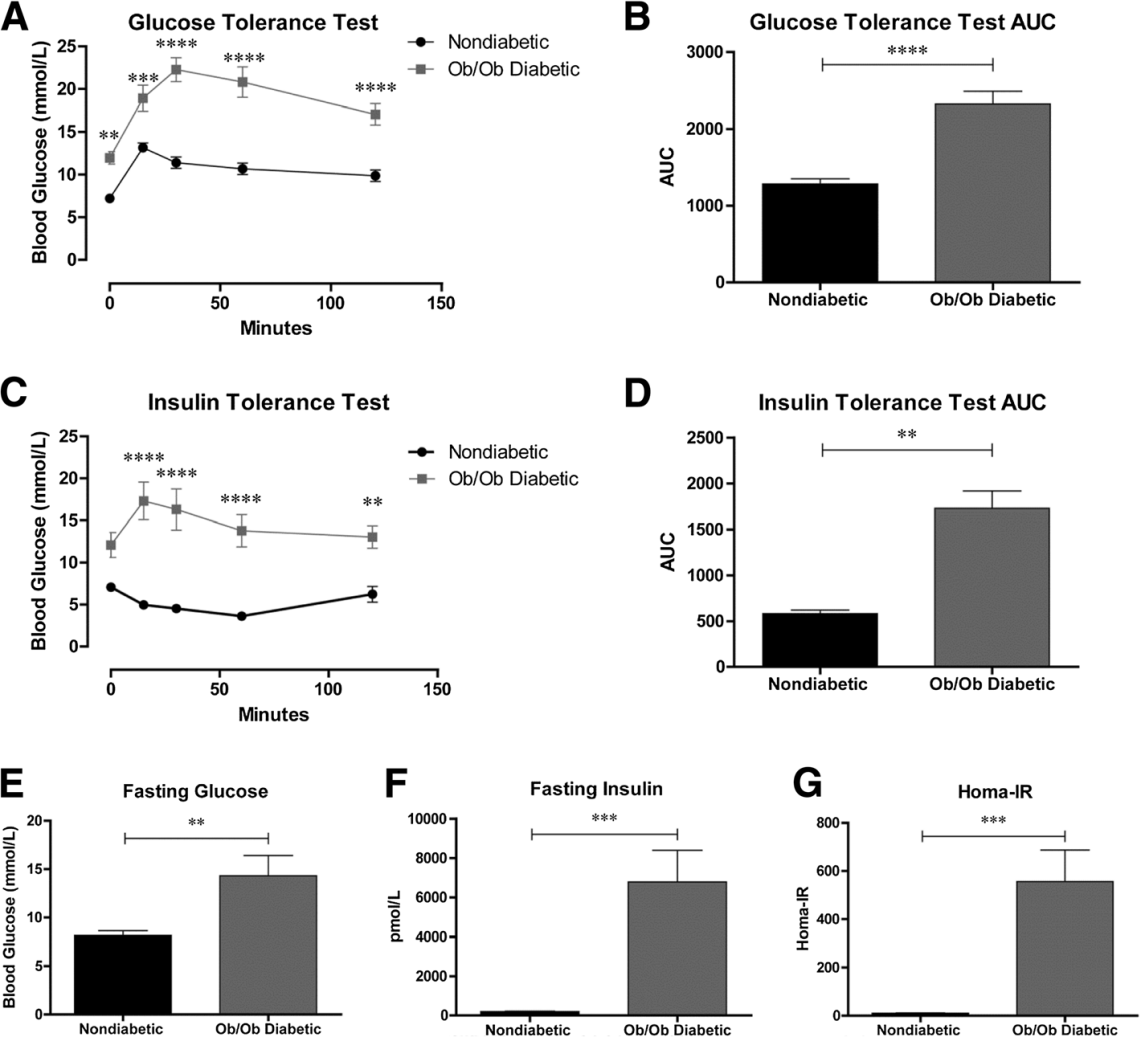
***Ob/ob *mice display classic signs of insulin resistance. A**, **B**) An IPGTT showed significantly elevated blood glucose levels in *ob/ob* mice throughout the test. The blood glucose of *ob/ob* mice increased more than 10 mmol/L at its maximal level as opposed to nondiabetic mice that elevated less than 6 mmol/L after glucose injection, indicating severe glucose intolerance in *ob/ob* mice. **C, D**) Similar to the IPGTT, data from the ITT showed reduced insulin sensitivity in *ob/ob* mice. In fact, an insulin dose of 1.5 U/Kg did not decrease the blood glucose level of *ob/ob* mice, whereas this dose lowered the blood glucose of nondiabetic controls by approximately 3.6 mmol/L. **E-G**) At 10 weeks of age, *ob/ob* mice had significantly elevated blood glucose and serum insulin levels. Accordingly, the HOMA-IR measure of insulin resistance was significantly higher in *ob/ob* mice. ** = p < 0.01, *** = p < 0.001, **** = p < 0.0001. IPGTT n = 7 nondiabetic mice, n = 6 *ob/ob.* ITT n = 4 nondiabetic mice, n = 4 *ob/ob.*

To quantify the extent of systemic insulin resistance in *ob/ob* mice, nondiabetic and diabetic *ob/ob* mice underwent an IPGTT at 9 weeks of age (Figure 1A). Blood glucose levels of the *ob/ob* mice were significantly higher than nondiabetic mice throughout the course of the experiment and the area under the curve (AUC) was also significantly elevated for *ob/ob* mice (Figure 1B). Results from the ITT indicated that nondiabetic, insulin-injected mice exhibited an expected physiological decrease in blood glucose in response to insulin; however, *ob/ob* mice displayed a transient elevation of glucose levels (Figure 1C). Statistical analysis of the data revealed that *ob/ob* mice maintained elevated glucose levels compared to nondiabetic controls throughout most of the study, and that the AUC was significantly higher for diabetic *ob/ob* mice (Figure 1D). The HOMA-IR, a measure of insulin resistance, was calculated using fasting blood glucose and serum insulin levels from 10 week old mice. *Ob/ob* mice had significantly higher blood glucose levels (14.3 ± 2.1 mmol/L) compared to nondiabetic mice (8.2 ±0.5 mmol/L) (Figure 1E). Fasting insulin levels were also significantly higher in diabetic *ob/ob* mice (6780 ± 1610 pmol/L) compared to nondiabetic mice (198 ± 25 pmol/L, Figure 1F). As such, *ob/ob* mice had a significantly elevated HOMA-IR as compared to nondiabetic mice (557 ± 130 compared to 10.1 ± 1.4, respectively, Figure 1G). These results demonstrate significant glucose intolerance and insulin resistance in *ob/ob* mice at this age.

### Mechanical allodynia in *ob/ob* mice

**Figure 2:**
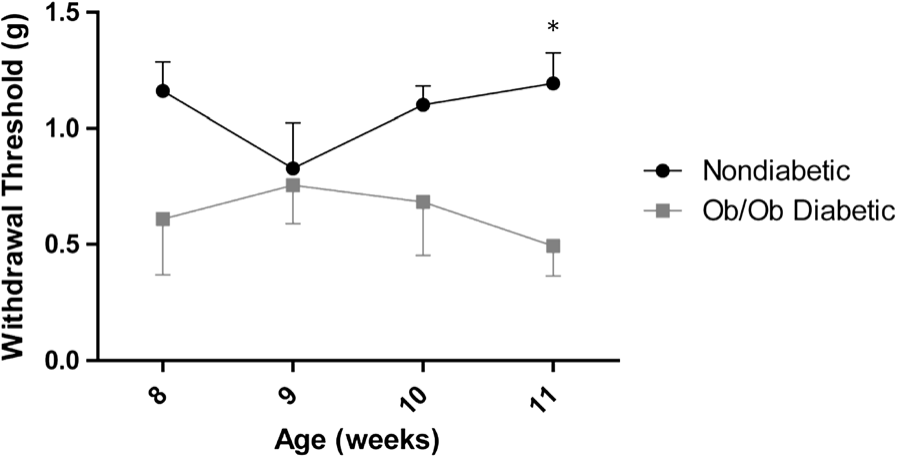
***Ob/ob *mice develop mechanical allodynia.** Mechanical thresholds were tested using von Frey monofilaments at 8, 9, 10, and 11 weeks of age. *Ob/ob* mice did not display significant differences from nondiabetic controls at 8, 9, or 10 weeks. However, at week 11, *ob/ob* mice had a significant decrease in their mechanical withdrawal thresholds. * = p < 0.05. n = 6 nondiabetic mice, n = 6 *ob/ob* diabetic mice.

To quantify a known behavioral abnormality associated with neuropathy in mice, mechanical sensitivity was assessed in nondiabetic and diabetic *ob/ob* mice at 8, 9, 10, and 11 weeks of age. There were no differences in mechanical thresholds between nondiabetic and *ob/ob* diabetic mice at 8, 9, or 10 weeks of age. However, at 11 weeks, there was a significant decrease in the mechanical thresholds of diabetic *ob/ob* mice compared to nondiabetic mice (Figure 2), consistent with sensory aberrations associated with peripheral neuropathy as previously reported in this genetic mouse strain [36].

### Blunted insulin and IGF-1 Akt activation in *ob/ob* DRG and sciatic nerve

Insulin stimulation causes a robust activation of Akt in insulin-sensitive tissues like muscle and adipose, as well as in neurons of both the peripheral and central nervous systems. Moreover, reduced insulin-induced activation of Akt is a hallmark of insulin resistance [5,6,37,38]. Here, nondiabetic and diabetic *ob/ob* mice were administered either intrathecal PBS or insulin and the DRG and sciatic nerve were harvested 30 minutes later for Western blot analysis to assess Akt activation. Both nondiabetic control and *ob/ob* mice display significantly elevated blood glucose levels following intrathecal injection of PBS. Nondiabetic mice glucose levels increased from 6.6 ± 0.4 mmol/L to 8.6 ± 0.5 mmol/L, whereas *ob/ob* levels increased from 12.1 ± 2.4 mmol/L to 22.3 ± 2.4 mmol/L. Glucose levels in nondiabetic mice significantly decreased from 7.0 ± 0.3 mmol/L to 3.3 ± 0.3 mmol/L after intrathecal insulin injection. *Ob/ob* mice glucose levels 30 minutes after insulin injection were not significantly different from baseline, starting at 12.7 ± 0.9 mmol/L and ending at 12.3 ± 1.5 mmol/L after 30 minutes.

**Figure 3:**
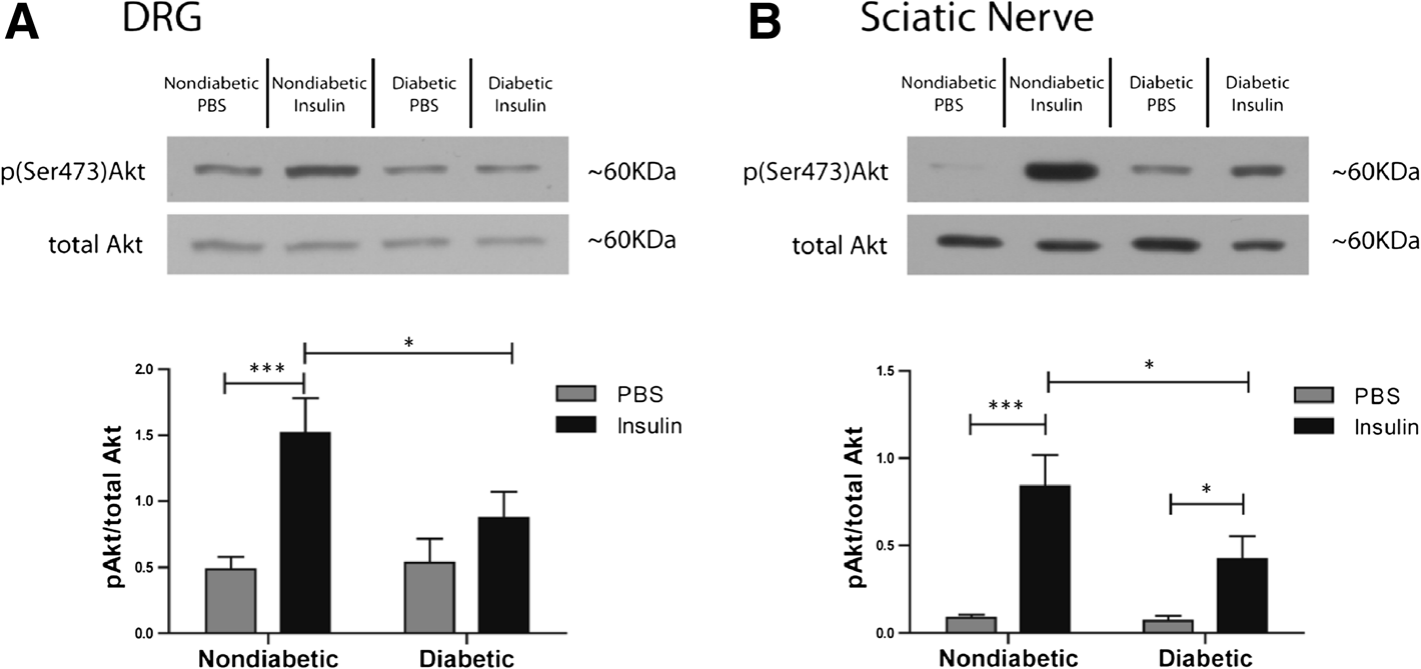
**Intrathecal insulin-induced Akt activation is blunted in the PNS of *ob/ob *mice.** DRG (**A**) and sciatic nerve (**B**) were harvested after an intrathecal injection of PBS (nondiabetic n = 10, *ob/ob* n = 7) or insulin (nondiabetic n = 10, *ob/ob* n = 9) was administered to nondiabetic control and *ob/ob* mice. Nondiabetic mice displayed a robust and significant increase in Akt activation with insulin stimulation; however insulin failed to significantly activate Akt in the DRG of *ob/ob* mice. Furthermore, the maximal increase in Akt activation with insulin stimulation was significantly lower in both the DRG and sciatic nerve of *ob/ob* mice. There were no differences in mice that received PBS in either the DRG or sciatic nerve. * = p < 0.05, *** = p < 0.001.

In nondiabetic mice, insulin produced a strong elevation in levels of activated Akt (p(ser473)Akt/total Akt) in both the DRG and sciatic nerve (Figure 3A,B). However in *ob/ob* mice, Akt activation was significantly lower in the DRG and sciatic nerve. In fact, insulin failed to significantly increase Akt activation over baseline in the DRG of *ob/ob* mice. For comparison, Akt activation in the DRG was increased 3.1 fold in nondiabetic mice and 1.6 fold in *ob/ob* diabetic mice. In the sciatic nerve, insulin produced 9.7 and 6.1 fold increase in Akt activation in nondiabetic and *ob/ob* mice, respectively.

**Figure 4:**
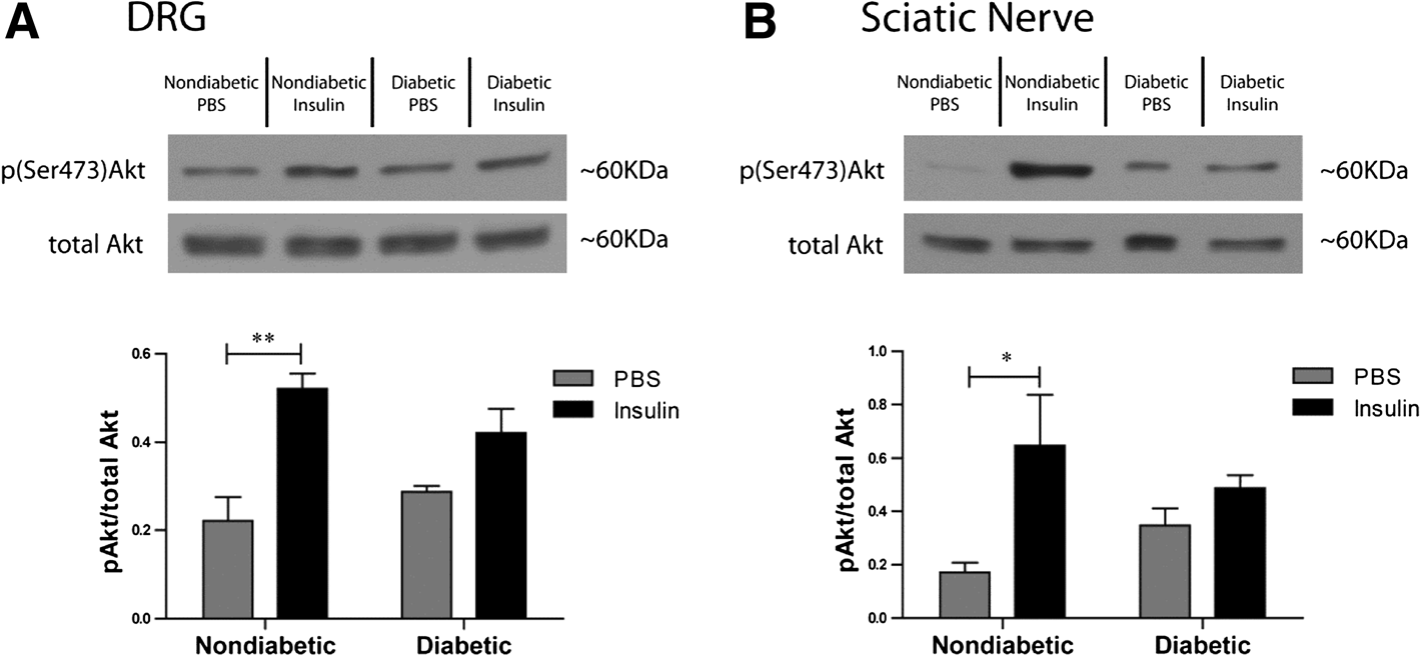
**The PNS of *ob/ob *mice showed reduced insulin-induced Akt activation in response to intraperitoneally-delivered insulin.** Nondiabetic and *ob/ob* diabetic mice were given intraperitoneal injections of PBS (nondiabetic n = 3, *ob/ob* n = 3) or insulin at a dose of 3.33 U/kg (nondiabetic n =3 and *ob/ob* n = 3). In both the DRG (**A**) and sciatic nerve (**B**) of nondiabetic mice, there was a significant increase in Akt activation in the insulin stimulated group as compared to mice that received PBS, yet no statistically significant changes were observed in the PNS from *ob/ob* mice. * = p < 0.05, ** = p < 0.01.

To confirm that these results were not dependent on the intrathecal route of delivery, a small number of mice were administered intraperitoneal insulin at a dose of 3.33 U/kg. PBS injections once again appeared to cause an increase in blood glucose levels from baseline, nondiabetic mice levels started at 7.0 ± 1.4 mmol/L and ended at 8.6 ± 1.4 mmol/L (p > 0.05), whereas *ob/ob* mice showed a significant increase from 10.8 ± 0.8 mmol/L to 15.4 ± 1.0 mmol/L. IP insulin resulted in significantly lower blood glucose levels in nondiabetic mice after 30 minutes, 7.8 ± 0.6 versus 4.2 ± 0.5 mmol/L, respectively. *Ob/ob* mice blood glucose levels were not significantly altered by IP insulin injection 15.3 ± 3.0 versus 13.6 ± 3.9 mmol/L. Similar to the intrathecal delivery route, a significant increase in Akt activation was observed in the DRG and sciatic nerve of nondiabetic mice stimulated with insulin; however, no significant change was observed in either tissue from *ob/ob* mice. (Figure 4A,B). In the DRG, nondiabetic mice displayed a 2.4 fold change in Akt activation, compared to a 1.5 fold change in *ob/ob* mice. IP insulin induced a 3.8 fold change in Akt in the sciatic nerve of nondiabetic mice, but only a 1.4 fold change in *ob/ob* in the sciatic nerve from *ob/ob* mice*.*

**Figure 5:**
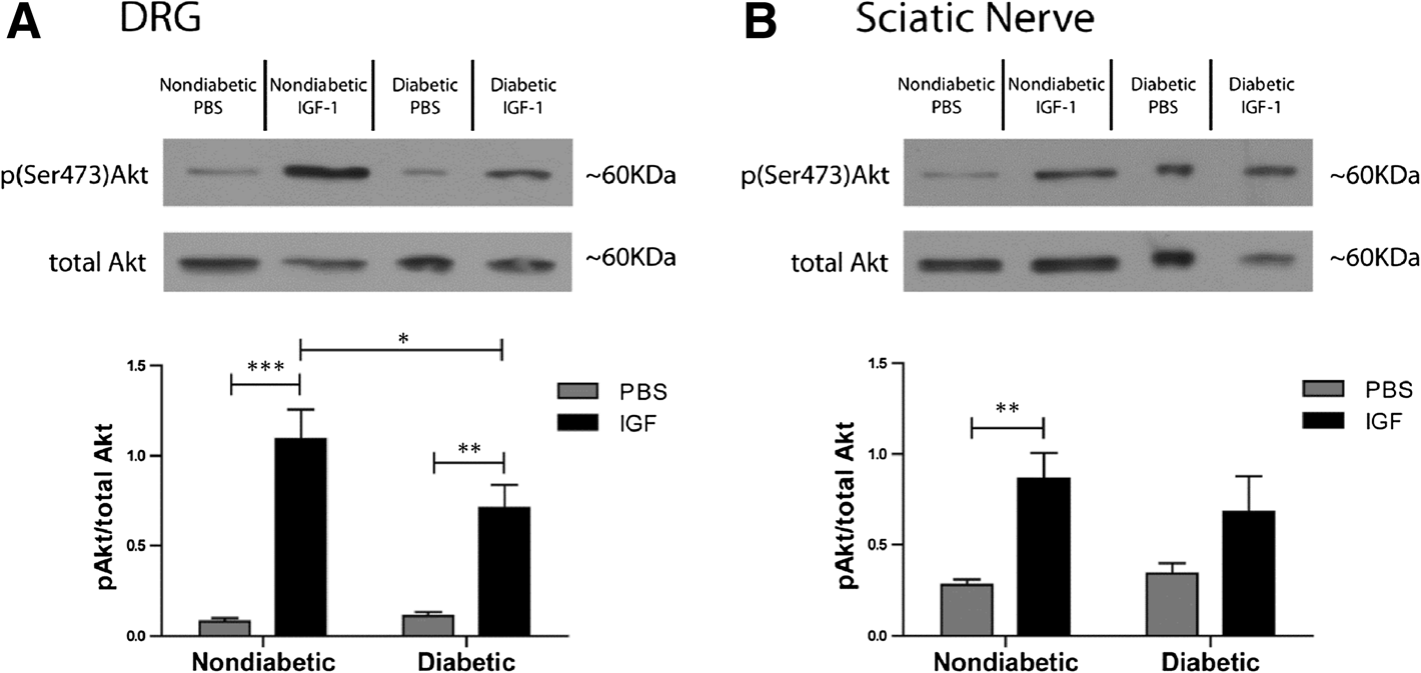
**The PNS of *ob/ob *mice displayed reduced Akt activation in response to intrathecal IGF-1.** Similar to the results shown for intrathecal insulin, an intrathecal injection of IGF-1 produced a strong activation of Akt in both the DRG and sciatic nerve of nondiabetic mice, but the response was somewhat blunted in the PNS of *ob/ob* mice. In the DRG (**A**), there was a significant increase in Akt activation in both the nondiabetic and *ob/ob* mice; however, the activation level was significantly lower in the DRG from *ob/ob* mice. In the sciatic nerve (**B**), IGF-1 stimulation resulted in a significant Akt activation in nondiabetic mice, but not in the *ob/ob* mice. * = p < 0.05, ** = p < 0.01, *** = p < 0.001. n = 8 nondiabetic PBS, n = 9 nondiabetic IGF-1, n = 7 diabetic PBS, n = 10 diabetic IGF-1.

IGF-1 and insulin activate many of the same intracellular signaling pathways [21], and altered IGF-1 signaling has been demonstrated in states of insulin resistance [39]. Furthermore, IGF-1 resistance has recently been demonstrated to be associated with brain insulin resistance and cognitive decline in Alzheimer’s patients [40]. To investigate IGF-1 signal transduction in the PNS of *ob/ob* mice, a dose of IGF-1 equimolar to 0.1U insulin was administered via an intrathecal injection. Blood glucose levels in both nondiabetic control and *ob/ob* mice once again appeared to increase with intrathecal PBS injection, 7.7 ± 0.3 mmol/L at baseline as compared to 9.7 ± 0.8 mmol/L (p = 0.056) after 30 minutes for nondiabetic mice and 12.1 ± 1.9 mmol/L at baseline to 23.7 ± 3.1 mmol/L after 30 minutes for *ob/ob* mice. IT IGF-1 did not significantly alter blood glucose levels in nondiabetic mice, 9.3 ± 0.4 mmol/L versus 8.1 ± 0.3 mmol/L. *Ob/ob* mice that received IT IGF-1 had similar blood glucose profiles to *ob/ob* mice that received IT PBS, with a significant increase in blood glucose after 30 minutes, 16.3 ± 2.7 mmol/L as compared to 27.7 ± 1.2 mmol/L. Akt was significantly activated in the DRG from both nondiabetic (13.3 fold) and *ob/ob* diabetic mice (6.0 fold). However, Akt activation was significantly lower in the DRG from *ob/ob* mice compared to nondiabetic mice (Figure 5A). In the sciatic nerve of nondiabetic mice, IGF stimulation produced a significant 2.8 fold increase in Akt activation. In contrast, Akt was not significantly activated in the sciatic nerve of *ob/ob* mice (Figure 5B).

### Insulin signaling downstream of Akt in the DRG and sciatic nerve

To assess whether diabetes-induced blunting of Akt activation was maintained downstream, several other insulin-responsive proteins were investigated via Western blot analysis, including mTor (protein synthesis), p70S6K (protein synthesis), AS160 (glucose uptake), and GSK3β (glycogen synthesis). At the insulin dose (0.1U) and time point (30 minute stimulation) that were investigated, no statistical differences (p > 0.05) were observed in the activation of these proteins even in control mice (Table 1). Additionally, similar to Akt results, no differences were observed in baseline levels between all proteins investigated. However, it is interesting to note that in both the DRG and sciatic nerve from *ob/ob* mice, there is a consistent pattern of a reduced fold change in response to insulin as compared to responses in nondiabetic mice.

**Table 1:** Downstream Akt pathway activation in the DRG and sciatic nerve after intrathecal insulin stimulation

**Protein of interest**	**DRG**		**Sciatic nerve**	
	**Control nondiabetic**	***ob/ob *** **diabetic**	**Control nondiabetic**	***ob/ob *** **diabetic**
	**Insulin-induced fold change**	**Insulin-induced fold change**	**Insulin-induced fold change**	**Insulin-induced fold change**
mTor	1.52	1.00	1.13	0.98
AS160	1.47	1.28	2.13	1.22
p70S6K	1.00	1.04	1.09	0.93
GSK3β	1.26	1.17	1.56	0.88

Four proteins downstream of Akt that are known to be involved in the intracellular actions of insulin signaling were investigated in the PNS of nondiabetic and *ob/ob* mice. In both the DRG and sciatic nerve, there were no significant changes in the activation of mTor, p70S6K, or AS160 in either nondiabetic or *ob/ob* mice, nor was there a significant change in the inhibition of GSK3β. Data presented is the fold change of protein modification (measured with Western blot analysis) induced by insulin as compared to that observed in mice that received PBS. n = 7-10 for all groups.

### The PNS of *ob/ob* mice display reduced insulin receptor expression and increased JNK activation

**Figure 6:**
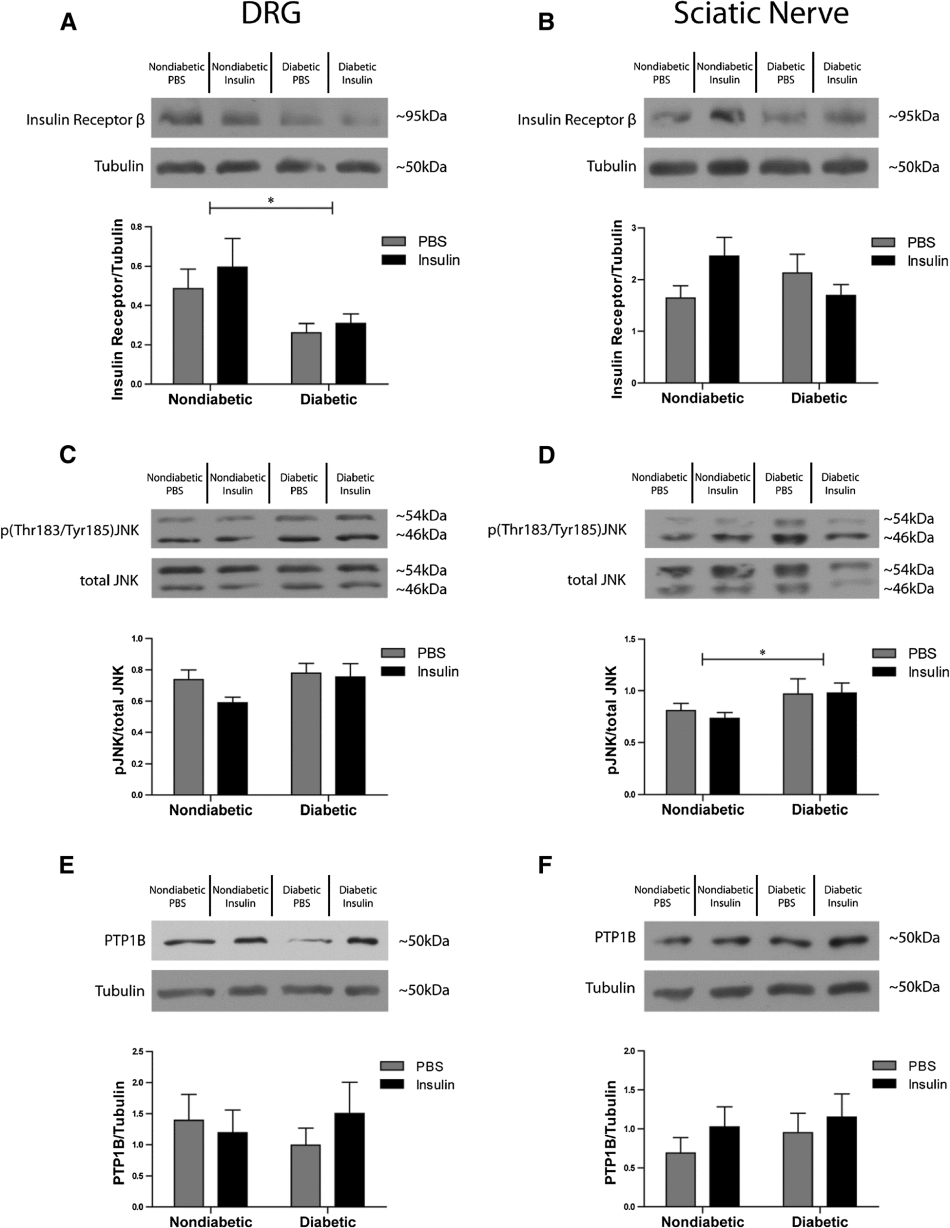
**Possible mechanisms that may be contributing to insulin resistance in the PNS. A**) In this study the expression of the beta subunit of the insulin receptor was significantly reduced in the DRG of *ob/ob* mice as compared to nondiabetic controls. **B**) No significant change in insulin receptor expression was observed in the sciatic nerve. **C**) The stress kinase JNK was not significantly activated in the DRG of *ob/ob* mice; however in the sciatic nerve (**D**) there was a significant upregulation of JNK in *ob/ob* mice. **E**, **F**) No differences in PTP1B expression profiles were observed in either the DRG or sciatic nerve between nondiabetic and diabetic groups. * = p < 0.05. n = 9 nondiabetic PBS, n = 9 nondiabetic insulin, n = 7 diabetic PBS, n = 9 diabetic insulin.

To explore possible mechanisms responsible for reduced PNS insulin sensitivity, we investigated several pathways known in other insulin-resistant tissues. One contributor to reduced insulin signaling is a downregulation of insulin receptor expression induced by hyperinsulinemia [23]. As shown in Figure 6A, protein levels of the insulin receptor subunit β were significantly lower in the DRG of *ob/ob* mice compared to nondiabetic mice. However, there was no statistical difference in the expression of insulin receptor between nondiabetic and *ob/ob* mice in the sciatic nerve (Figure 6B). No significant differences between groups were observed in IGF-1 receptor expression in either the DRG or sciatic nerve (data not shown).

Our previous studies in primary DRG cultures reported an upregulation of IRS2 serine phosphorylation [6], a recognized mechanism of insulin resistance in muscle and adipose. In the current study, we investigated both IRS1 (muscle and adipose isoform) [41] and IRS2 (neural isoform) [6,42] serine phosphorylation. In contrast to neurons *in vitro*, IRS serine phosphorylation does not appear to be significantly affected in the PNS *in vivo* within this model, (data not shown). Interestingly, there was significant activation of the stress kinase JNK (p(Thr183/Tyr185)JNK/total JNK) in the sciatic nerve of *ob/ob* mice compared to nondiabetic mice (Figure 6D) and a similar pattern of activated JNK was observed in the DRG of *ob/ob* mice, however significance was not reached (nondiabetic vs. diabetic p = 0.122) (Figure 6C).

In addition to stress kinase activation and reduced insulin receptor expression, insulin resistance can also be induced by over activation of tyrosine phosphatases [29]. However, PTP1B expression was not elevated in the DRG or sciatic nerve of *ob/ob* mice, nor did insulin stimulation appear to alter its expression levels (Figure 6E,F, respectively).

## Discussion

Diabetic neuropathy is associated with profound loss of distal limb sensation and/or pain, causing significant decline in the quality of life and potential morbidity and mortality for patients. Currently, there are no clinical treatments that successfully improve neuropathic damage to peripheral sensory nerve fibers, likely due to the multifactorial etiology of neuropathy development and progression. Here, we have demonstrated *in vivo* PNS insulin resistance in *ob/ob* mice. These results are consistent with recent *in vitro* studies and support the view that altered insulin signaling may contribute to DN [43]. A robust activation of insulin-sensitive pathways was observed in the DRG and sciatic nerve of nondiabetic mice, with a blunted response in both tissues from insulin-resistant *ob/ob* mice. While no one mechanism of insulin resistance was clearly prevalent, significant changes were seen in two known pathways of insulin resistance, including increased JNK activity and reduced insulin receptor expression. Although more research is needed to fully elucidate the pathways leading to PNS insulin resistance, these results suggest that cellular mechanisms of insulin resistance that have been defined in muscle may also play an important role in the PNS.

These experiments used an *in vivo* approach to support the mounting *in vitro* evidence pointing to PNS insulin resistance in diabetes. Interestingly, Akt activation was very prominent in the DRG and sciatic nerve of nondiabetic mice, yet very few significant changes were seen in downstream signaling molecules. This may be due to a temporal effect, as downstream mediators of the Akt pathway may have not yet been activated during the 30-minute stimulation period used for this study. However, it is also plausible that the downstream Akt signaling proteins explored in this study do not play a prominent role in insulin pathways within the DRG. Instead of driving protein synthesis through mTor and p70S6K or regulation of GSK3β actions, insulin may be playing a more important role in lipid and glucose metabolism, gene regulation, or mitochondrial maintenance in peripheral neurons. Further studies are underway to explore these other downstream components of insulin signaling and to define the temporal components of this signaling pathway.

An additional caveat to this study is the use of leptin-deficient *ob/ob* mice. Leptin’s role in the nervous system is receiving increasing attention, and it may have a neuroprotective role [44]. It is not known how reduced neuronal leptin may have contributed to our results. Thus, confirming these results in a high-fat diet model of obesity will be an important step to further investigating PNS insulin resistance.

In experiments presented here, it appeared that insulin produced a stronger Akt activation in the sciatic nerve compared to the DRG (Figure 3), whereas IGF-1 produced a stronger Akt activation in the DRG compared to the sciatic nerve (Figure 5). These results point to an apparent separation in insulin/IGF-1 signaling support within the PNS. One plausible explanation may be that insulin and IGF-1 have different actions on the DRG soma and satellite cells compared to sensory axons, motor axons, and Schwann cells in the peripheral nerve, leading to alternative signaling profiles. How this potential divergence in signaling may affect sensory neuron function is yet to be determined and ongoing research is further delineating the differential roles that insulin and IGF-1 may play in sensory nerve biology.

In *ob/ob* mice, both the DRG and sciatic nerve displayed reduced insulin-induced Akt activation, a classic indication of insulin resistance. Several mechanisms of insulin resistance outlined in muscle also appear to be altered in the PNS, and may be contributing to the observed reduction in insulin signal transduction. However, these results must be interpreted with caution as significant changes were not seen consistently across PNS tissues, and further research will need to be completed to fully establish a clear mechanism. Interestingly, no change in baseline Akt activation levels was observed between nondiabetic and *ob/ob* mice as may be expected in states of insulin resistance. These results are intriguing and suggest that future research focusing on pathways driving Akt signaling is warranted.

Hyperinsulinemia can promote insulin resistance through downregulation of the insulin receptor [22]. This effect was demonstrated in our data. The *ob/ob* mice in this cohort had serum insulin levels 34.3 fold higher than nondiabetic mice and the DRG of *ob/ob* mice displayed significantly lower insulin receptor expression. Thus, the extreme hyperinsulinemia in the *ob/ob* mice may be promoting insulin receptor downregulation and contributing to PNS insulin resistance. This idea is supported by a recent study that reported a significant decrease in insulin receptor mRNA in cultured DRG neurons that displayed insulin resistance when treated with high levels of insulin [7].

An alternative mediator of insulin resistance is the stress kinase JNK, which is activated in response to various cellular stressors, including low grade chronic inflammation induced by obesity [33,45]. In fact, *ob/ob* mice with a JNK null mutation have improved whole body glucose tolerance and insulin sensitivity [31]. Additionally, JNK activation has been implicated in altered neurofilament phosphorylation in the PNS of diabetic rats [46]. JNK activation is proposed to promote insulin resistance through upregulation of IRS serine phosphorylation, and IRS is a key common signaling component of both the insulin and IGF-1 pathways. In the current study we observed increased JNK activation without a significant elevation in either IRS1 or IRS2 serine phosphorylation. Some controversy does exist as to which serine sites are most important in insulin resistance, thus the serine sites that we probed (p(ser731)IRS2 and (p(ser307)IRS1) may not be heavily involved in inhibiting insulin signaling in the PNS. More powerful approaches, such as mass spectrometry, may be needed to establish a global change in the IRS phosphorylation profile within the PNS [47].

Another possible component of the insulin receptor signaling pathway that could be affected in insulin resistance is PTP1B. PTP1B is the canonical member of protein tyrosine phosphatases and serves an important role in insulin signaling regulation [29]. Overexpression of PTP1B has been linked to insulin resistance in peripheral tissues of *ob/ob* mice [26] and PTP1B knockout mice display increased insulin sensitivity [48]. In the current study, we did not detect significant upregulation of PTP1B in the DRG or sciatic nerve of insulin resistant mice. While there was no change in PTP1B expression, there still could be alterations in phosphatase activity and further studies are underway to explore this possibility.

It will be important to put the current results in context with other contributory mechanisms of DN, including glucose and/or lipid mediated toxicity as well as oxidative stress [49]. We postulate that the metabolic dysfunction associated with hyperglycemia and dyslipidemia in concert with reduced neurotrophic support promotes deterioration and reduced regeneration of the distal axon. Furthermore, the loss of appropriate insulin signaling could make neurons even more susceptible to these pathogenic cascades. It should be noted that both intrathecal and intraperitoneal insulin injections altered blood glucose levels. Thus, these results should be viewed in the context that glucose levels were also transiently altered by insulin administration. Further research into disrupted PNS insulin signaling relative to other pathogenic mechanisms is needed, as this will be a key step in translating these basic science results into clinical applications.

## Conclusions

Insulin resistance is emerging as a potential mediator of several neurological syndromes (reviewed in [1]). This study, along with recent data of *in vitro* DRG insulin resistance, strongly supports altered insulin signaling as a pathogenic mechanism in DN. While deficient insulin signaling has been a proposed contributor to DN in type 1 models for some time [3,4,14,43,50], little has been known about insulin signaling effectiveness in type 2 (hyperinsulinemic) models of DN. We observed reduced insulin signaling *in vivo* in the PNS of type 2 diabetic *ob/ob* mice and suggest possible mechanisms that may be contributing to these changes. It is now becoming evident that decreased insulin neurotrophic support in the PNS is an integral part of DN and may be a congruent mechanism between type 1 and type 2 diabetic models of DN, as both have reduced insulin signaling either due to insulinopenia or neuronal insulin resistance.

Future studies will focus on mechanisms through which insulin supports proper PNS function, as revealing these pathways may provide insight into how decreased insulin support contributes to the pathogenesis of DN. Furthermore, delineating the details of PNS insulin signaling may open new avenues for therapeutic intervention in patients with DN.

## Methods

### Animals

All experiments were approved by the University of Kansas Medical Center Institutional Animal Care and Use Committee. Male *ob/ob* leptin null mutant and age-matched control mice (*ob/+*) were purchased from Jackson Laboratories (Bar Harbor, Maine) at 8 weeks of age. Mice were given access to food and water ad libitum and housed on a 12-hour light/dark cycle. Weekly blood glucose (Glucose Diagnostic Assay Sigma-Aldrich, St. Louis, MO), serum insulin (Insulin ELISA Alpco, Salem, NH) and weights were monitored and mice were sacrificed at 11 weeks of age.

### Glucose tolerance test

At 9 weeks of age, an intraperitoneal glucose tolerance test (IPGTT) was used to assess the response of mice to a glucose challenge. After a 6-hour fast, mice were given an intraperitoneal injection of glucose at 1g of glucose per kg body weight. Blood glucose levels were measured via tail clip immediately prior to the glucose bolus and then at 15, 30, 60, and 120 minutes after injection.

### Insulin tolerance test

At 10 weeks of age, mice underwent an insulin tolerance test (ITT). Mice were fasted for 6 hours and then administered IP insulin (Humulin R, Lilly, Indianapolis, Indiana) at a dosage of 1.5 U per kg body weight. Blood glucose levels were monitored immediately prior to insulin injection and then at 15, 30, 60, and 120 minutes thereafter.

### HOMA-IR

Fasting insulin and fasting glucose levels were used to calculate the homeostatic model assessment of insulin resistance (HOMA-IR). Scores were calculated with the following equation: (blood glucose (mg/dl) X (serum insulin (uU/mL))/405) [51].

### Mechanical sensitivity

Mechanical behavioral responses to Semmes Weinstein-von Frey monofilaments (0.07 to 5.0 g) were assessed at 8, 9, 10, and 11 weeks of age. Mice underwent acclimation 2 days prior to the first day of behavioral testing. Mice were placed in individual clear plastic cages (11×5×3.5 cm) on a wire mesh grid 55 cm above the table and were acclimated for 30 minutes prior to behavioral analysis. The filaments were applied perpendicularly to the plantar surface of the hindpaw until the filament bent. Testing began with the 0.7 g filament, and in the presence of a response, the next smaller filament was applied. If no response was observed, the next larger filament was used. Filaments were applied until there was a change in response, followed by an additional 4 more applications. The withdrawal threshold was calculated using the formula from the up-down method previously described [52].

### Insulin and IGF-1 injections

Sterile PBS (vehicle), 0.1U (~0.7 nmol) Humulin R insulin, or recombinant IGF-1 equimolar to 0.1U insulin was directly administered to both nondiabetic and *ob/ob* type 2 diabetic mice via a one-time intrathecal injection. Previously, intrathecal 0.1U insulin and equimolar IGF-1 have been shown to have beneficial effects on the symptoms of DN [10]. All injections were 50 μL and administered with a 1cc 28½ gauge insulin syringe between the L6 and S1 vertebrae. In an additional preliminary study, sterile PBS or insulin was delivered through an intraperitoneal injection at a dose of 3.33 U/kg, such that the total insulin administered was approximately 0.1 U for nondiabetic mice and 0.17U (~1.2 nmol) for *ob/ob* mice. The doses administered and stimulation time frames used were confirmed to be sufficient for Akt activation in the PNS with dose curve and time course studies (Grote, unpublished observation).

### Western blots

After a 30 minute insulin stimulation period, the right and left lumbar DRG and sciatic nerves were harvested for each sample from 11 week old mice and frozen at −80°C. Tissues were sonicated in Cell Extraction Buffer (Invitrogen, Carlsbad, CA) containing 55.55 μl/ml protease inhibitor cocktail, 200 mM Na_3_VO_4_, and 200 mM NaF. Following sonication, protein was extracted on ice for 60 minutes and vortexed every 10 minutes. After centrifugation, protein concentration of the supernatant was measured with a Bradford assay (Bio-Rad, Hercules, CA). Samples were then boiled with Lane Marker Reducing Sample Buffer (Thermo Scientific, Waltham, MA) for 3 minutes. Equal amounts of protein (30 μg) were loaded per lane and samples were separated on a 4-15% gradient tris-glycine gel (Bio-Rad), and then transferred to a nitrocellulose membrane. Membranes were probed with the following primary antibodies and all antibodies were purchased from Cell Signaling (Danvers, MA) unless otherwise noted: total Akt (1:2000), p-(Ser473)Akt (1:500), total p70S6K (1:500), p-(Thr389)p70S6K (1:500), total GSK3β (1:1500), p-(Ser9)GSK3β (1:1000), total JNK (1:1000), p-(Thr183/Tyr185)JNK (1:500), total mTor (1:500), p-(Ser2448)mTor (1:500), Insulin-like growth factor 1 receptor β subunit (1:500), PTP1B (1:500) (Abcam, Cambridge, MA), total AS160 (1:1000) (Millipore, Billerica, MA), p-(Thr642)AS160 (1:500) (Millipore), Insulin Receptor β subunit (1:500) (Santa Cruz, Santa Cruz, CA), and α-tubulin (1:5000) (Abcam). Bands were visualized with either anti-mouse or anti-rabbit HRP-conjugated secondary antibodies (Santa Cruz) and ECL with X-ray film. Densitometry with ImageJ (NIH) was then used to analyze each lane. All samples from each tissue were run simultaneously across multiple gels and each group was equally represented on each gel (approximately 3 nondiabetic PBS, 3 nondiabetic insulin, 3 *ob/ob* PBS, and 3 *ob/ob* insulin per gel). Data is presented as the ratio of integrated density of the phosopho-protein normalized to the integrated density of the total protein. The normalized ratio was averaged for each group and the mean ± SEM is represented in the corresponding figures. Representative immunoblots are shown.

### Statistical analysis

All data is expressed as means ± standard error of the mean. IPGTT, ITT, and behavior data were analyzed with a repeated measures analysis of variance (RM-ANOVA). In addition, the area under the curve (AUC) for IPGTT and ITT was analyzed using a Student’s *t*-test. Blood glucose changes at 30 minutes in response to insulin or IGF-1 were analyzed with a paired Student’s *t*-test. Western blot results were analyzed with 2-way ANOVA and Bonferroni’s post hoc analysis when appropriate. Outliers greater than or less than 2 standard deviations from the mean were not included in the analysis. All statistical tests were performed using SigmaPlot software and a *P* value <0.05 was considered significant.

## Abbreviations

CNS: Central nervous system; PNS: Peripheral nervous system; IRS: Insulin receptor substrate; mTor: Mammalian target of rapamycin; GSK3β: Glycogen synthase kinase 3 beta; MAPK: Mitogen-activated protein kinase; PTP1B: Protein tyrosine phosphatase 1B; JNK: c-Jun N-terminal kinase; DN: Diabetic neuropathy

## Competing interests

The authors have no competing interests to report.

## Authors’ contributions

CG carried out the Western blot analysis, participated in metabolic characterization of mice, performed the statistical analysis and drafted the manuscript. AG carried out the behavior analysis of mechanical sensitivity. JR performed intrathecal injections, tissue dissections, and participated in metabolic characterization of mice. PG helped conceive the study and participated in the design of the study. EF participated in data interpretation and experimental design and helped draft the manuscript. DW conceived the study, and participated in its design and coordination and helped to draft the manuscript. All authors read and approved the final manuscript.
